# Intensive Care Unit Nurses’ Perceptions of and Coping Strategies for Motherhood Experiences: A Qualitative Study

**DOI:** 10.3390/healthcare10091660

**Published:** 2022-08-31

**Authors:** Hui-Chuan Wu, Yueh-Chu Peng, Hsiu-Hung Wang

**Affiliations:** 1 College of Nursing, Kaohsiung Medical University, Kaohsiung 807, Taiwan; 2 Taichung Veterans General Hospital, Taichung 407, Taiwan

**Keywords:** motherhood experiences, work–family conflict, intensive care unit nurses, working women, qualitative study

## Abstract

This qualitative study explored the perception of intensive care unit nurses in relation to their motherhood experiences and coping strategies. Ten intensive care unit nurses (aged 28–37 years) with children under 3 years old were recruited. In-depth 90-min interviews were conducted, and the contents of the audio recordings were translated into verbatim transcripts. From the results of the research analysis, three themes were identified: challenges and conflicts of motherhood, dilemma in life and work balance, and maternal engagement. The “challenges and conflicts of motherhood” and “dilemma in life and work” represent the maternal experiences, while “maternal engagement” is the coping strategy used. To establish a friendly working environment and policies, nursing managers should consider improving the friendliness of work units and encourage co-workers to support each other in the hospital.

## 1. Introduction

The difficulty in balancing family and employment faced by women has been identified as one of the factors affecting the fertility rate in Taiwan [1]. In Taiwan, the peak working age of women is 25–29 years, with the labor participation rate reaching 90.47%. At 30–34 years of age, the period of marriage and childbearing, the proportion of labor begins to decline, falling to approximately 76.23% at 35–49 years. Generally, the labor participation rate of married women is approximately 24%, which is lower than that of unmarried women [2]. This suggests that marriage and childbearing affect the original lifestyle of women in the workplace and may make it difficult for them to balance work and family life simultaneously. Therefore, the parental role of professional women may exert a relatively heavy pressure on them.

Work pressure is more likely to affect the physical and mental states of women than that of men [3]. When the work pressure experienced by working women increases, role conflict pressure also increases, making them prone to a negative health state [4]. Parenting is difficult for both mothers and fathers, and it can be compounded by their employment situation, especially for women [5]. Changes in parental role pressure affect the work, family, and marriage balance of working women and further influence their self-image and self-shaping. To effectively improve the balance between work and family life, the Taiwanese government has been committed to promoting the Act of Gender Equality in Employment [6]. The government promotes various employment equality measures, such as providing nursing rooms at workplaces, so that breastfeeding working women get two 30-min sessions to expressing milk during work [6].

Breastfeeding is central to new mothers providing parental care. When women resume work after giving birth, besides continuous breastfeeding, they face various situational problems and physical and mental stress related to the parenting process. Previous studies on professional women mostly focused on how to build a breastfeeding-friendly workplace, the impact of a friendly breastfeeding workplace on women, work pressures, physical and mental burdens, and family conflict and role strain [4,7,8]. The role of being a mother and forming an attachment with children is a learning process that requires readjustment, despite inevitable maternal pressure while learning infant care skills and acquiring knowledge [4].

Clinical nursing work in hospitals requires swing shifts and nursing personnel have to bear high risks and work pressure [9]. Literature review studies related to overwork in ICUs have found that the work environment and nature of ICUs are different from those of the general ward [10]. As patients’ conditions are more critical in ICUs than in general wards, burnout of professional workers in ICUs is common [10]. While performing stressful work, the nursing personnel working in the ICU are prone to physical and mental exhaustion, although the signs of physical and mental wear and tear are not easily detected [11,12]. These may affect the quality of patient care, patient safety, and the ICU nursing personnel’s quality of life [13]. Previous studies have seldom focused on hospital clinical nurses’ adaptation and adjustment to the parental role, especially those of nursing personnel working in ICUs. What are the experiences of hospital nursing personnel in adapting to work and parenting? How do they perceive and cope with both? These are questions worthy of exploration. Since 2012, the Ministry of Health and Welfare has actively promoted the Nursing Reform Project [14]. Understanding the nursing workplace from the perspective of nursing personnel can help to build a better nursing workplace environment.

This study employed a qualitative and in-depth approach to investigate ICUs nurses’ work and coping experiences in relation to their parental role, which can be considered to create and promote a friendly nursing workplace environment.

## 2. Methods

### 2.1. Design

This study conducted qualitative research and used face-to-face in-depth interviews to obtain participants’ perceptions and experiences of motherhood.

### 2.2. Participants and Settings

Purposive sampling was used to recruit participants from a medical center in central Taiwan. The inclusion criteria were nurses who (1) worked in the ICU, (2) had children under 3 years old, and (3) consented to participate in the study. Ten ICU nurses (aged 28–37 years) from the medical center were recruited in this study, and each interview was conducted in a private room.

### 2.3. Data Collection

The investigator invited the nurses in-person from the departments that met the inclusion criteria to participate in the study. The time and venue for the formal interview were agreed upon after confirming with the participant that they were willing to participate. Before starting the interviews, the research procedure was explained to the participants, and consent was obtained. The participants could terminate participation at any time, and they were assured that their data would be kept confidential and used for research purposes only. All participant information was de-identified to protect their privacy. Semi-structured interviews of 60–90 min were conducted for each participant. The interview process was recorded, and then, transcribed verbatim for analysis.

### 2.4. Data Analysis

This study adopted the content analysis method to systematically analyze and code the interview text. After repeated inspections, according to the research purpose and semi-structured interview guidelines, similar codes were classified into a category, and different categories with related potential meanings were classified into the same theme [15].

### 2.5. Rigorousness

Regarding the trustworthiness of the research content, this study employed the principles proposed by Lincoln and Guba [16], including credibility, dependability, confirmability, and transferability. Regarding qualitative research methods, the principal investigator and co-investigators who had expertise in qualitative research and experiences in working in ICUs and with motherhood, acted as research tools. During the research implementation and data analysis process, discussions were continuously conducted with experts proficient in women’s research to improve the credibility and dependability of content analysis. All raw data, interview recordings, verbatim manuscripts, and analysis procedures, including the writing and complete coding of reflective records, and the process of categorization, were retained as bases for future academic review. Further, the text data and analysis categories were reviewed by the investigators; the implications of the data were clarified, and the confirmability of the study was ensured. This study was evaluated by providing a comprehensive description of the study that fits with other contexts. Therefore, transferability was established. Moreover, to enhance the investigators’ reflection during analysis, peer review was conducted. Peer review aids in detecting and precluding personal subjective experiences that may be involved in data analysis and the resulting interpretation, thus avoiding errors and increasing the rigor of this study [17]. From the participants’ interviews, the research purpose of this study could be fully achieved, and data saturation could be reached. The participants’ data were sorted, examined, and analyzed.

### 2.6. Ethical Consideration

The study protocol was reviewed and approved by Taichung Veterans General Hospital Human Ethics Review Board (TCVGH No. C09133).

## 3. Results

In this study, in-depth interviews of 10 participants were conducted. The demographic data of the participants are presented in Table 1.

**Figure 1:**
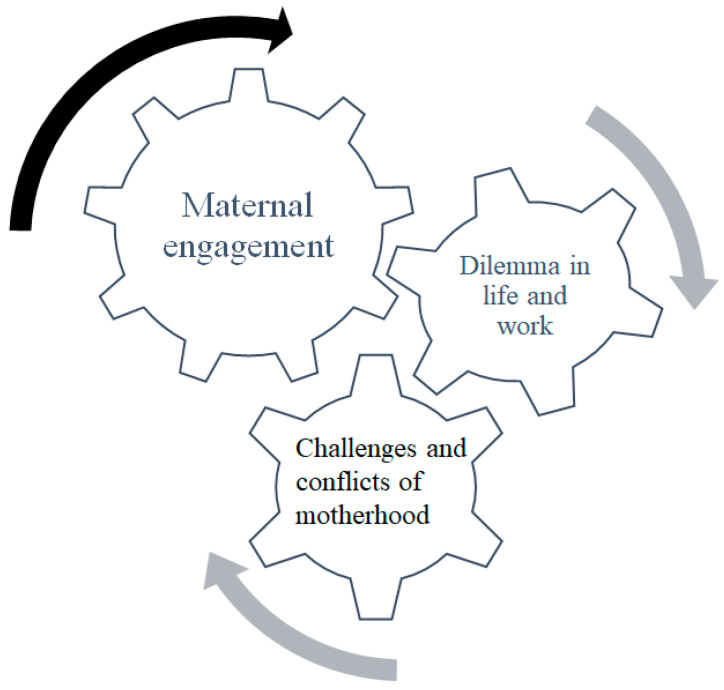
ICU nurses’ motherhood experiences and coping strategies.

The analysis revealed that ICU nurses’ motherhood experiences include the challenges of motherhood and the dilemma of family–work balance. Accordingly, there is a need for coping strategies to assist the nurses with role adaptation and to achieve a family-work balance. These three themes often interrelate with each other in a loop, that is, as motherhood challenges arise, coping strategies continue to be applied and play a role in the mothering process (Figure 1).

### 3.1. Theme 1: Challenges and Conflicts of Motherhood

Due to the participants’ nursing background, their family members assumed that they were capable of caring for infants and young children, and would find mothering easy and natural. However, they did not realize that the participants could not use their professional skills of infant care until they became mothers. Their family members’ methods of assisting in infant care lacked scientific basis; however, the participants were unable to dissuade them, and thus, felt challenged and conflicted.

#### 3.1.1. Between Professional and Motherhood Roles

Nursing does not equate to having knowledge of a mother’s role, meaning that even with a professional medical background, nursing personnel still may be unfamiliar with childcare.

“*By following the nursing routine, there will be no problem. I can’t take care of my own child with the nursing routine, so it is very problematic.*”

(No. 1)

“*When we first caught a cold, we were quite flustered, because although we were medical staff, we were new mothers and didn’t know much about caring for a child.*”

(No. 3)

“*When the child starts to cry from discomfort, he (the husband) will lose his temper. He thought that I didn’t dress him or change his clothes, how could the child accidentally catch a cold?*”

(No. 6)

Nurses’ knowledge of the nurse–client relationship does not extend to the parent-child relationship or family relations. While nursing is a symbol of caring, it does not imply parenting.

#### 3.1.2. Between Old and New Generation Ideas of Motherhood

The phenomenon where the elders intend to take control of everything is still common in many extended families.

“*I experience pressure from my mother-in-law mainly because she insists on using some folk therapies. She always tells me about how she raised my husband or brother-in-law. From my perspective, her way of child-raising was less scientific…*”

(No. 6)

The participants intended to rear children in their own way without being affected by the old habits and attitudes of the previous generation.

“*My mother-in-law thought it was because of breastmilk that the baby had diarrhea. She wants to feed formula milk. I let the baby drink formula milk, otherwise she (mother-in-law) will think it’s all my fault…*”

(No. 5)

Chinese society has an inherent culture of “obedience to elders.” When the older generation assists the new generation with parenting, new mothers loathe agreeing to their traditional or baseless parenting styles, giving rise to dilemmas of parenting differences across generations.

### 3.2. Theme 2: Dilemma in Life and Work

As the patients in an ICU are in critical condition, it is difficult for nurses to take an urgent leave. In addition, they are required to work shifts. These factors made it difficult for the participants to strike a work–family balance.

#### 3.2.1. Difficulty in Exclusive Breastfeeding While at Work

Breastfeeding is often regarded by postpartum mothers as a manifestation of motherhood. Therefore, even if they are tired and busy, they insist on breastfeeding during work. The participants were concerned that pathogenic bacteria in the ICU environment would affect the quality of their stored breastmilk and their child’s health.

“*Because I have no way to breastfeed, I can’t maintain breastfeeding, and I have to squeeze the breastmilk out during work. Therefore, I feel I’m being rushed at work. I will be busy completing my work, and then, squeeze my breastmilk in a hurry. However, I am worried about infection because ICU nurse practitioners are frequently exposed to isolated patients.*”

(No. 6)

The conditions of patients in the ICU are critical and often fluctuate. Although breastfeeding nurse practitioners have the right and time to express breastmilk at work, they inevitably worry about their work while doing so, which causes stress during work and while expressing breastmilk.

“*Our meeting room is near beds No. 17 and 18. I remember that…when I was squeezing breastmilk, the ventilator at these beds was beeping. I was busy squeezing breastmilk, and I worried about the beeping noise and felt extremely stressful. Fifteen minutes later, my colleague came to knock on the door and asked me why the ventilator was beeping. I had to wrap up immediately and go out to do my work.*”

(No. 9)

While some ICU colleagues supported nurses in expressing breastmilk while at work, others did not.

“*I feel that the policy does not match the actual situation. It is true that the policy has been formulated to support breastfeeding. However, not everyone will support it because it is impossible to sacrifice the rights of the other eight people at work so that you can collect breastmilk… It is unfair for other nurse practitioners to take care of more patients simply because you have to collect breastmilk. Besides, this also causes a bad feeling for other colleagues. What’s really important is whether the colleagues are willing to provide you with actual support.*”

(No. 10)

#### 3.2.2. Shift and Inflexible Work Conflict with Childcare

The course of patients in the ICU changes rapidly, with generally insufficient manpower and deployment. Nurses working there need support or extended working hours. They often need to focus on work and sacrifice their family life.

“*Sometimes, maybe a patient’s condition changes suddenly, like needing ECMO (extracorporeal membrane oxygenation)! Your working hours will be extended for a long time, yes! Even your vacation will get canceled… yes! You’re just going to go to work…*”

(No. 3)

“*If the work unit asks you to go to work, there is the pressure of who can take care of the children…yes! Because you have no way…you can’t just pass your child to other people, then you will have to take leave, right! The pressure will be more…it will feel more stressful*”

(No. 2)

If working nurses are unable to take temporary leave to care for their sick children, they feel sad and stressed.

“*I feel that…when your own child is sick and you have to go to work to take care of other people’s children, I really struggle and wonder why I am doing this. Why aren’t I taking care of my own child who is ill?*”

(No. 1)

In addition, swing shifts at work affect the parenting method. After the participants became mothers, they perceived the impact of swing shifts on parenting.

“*I am growing older, so I feel physically incompetent to work night shifts. My physical strength is probably reducing. The second reason may be that I am now raising a child, so it’s difficult for me to work night shifts…*”

(No. 9)

Resulting from the problem of manpower deployment in clinical work, the difficulty in taking temporary leave to care for their sick children distresses working mothers.

“*There will be conflicts, probably when the children are sick, they need someone to take care of them, but because of your own occupational problems, you cannot ask for leave at any time, and the burden will be a little bigger.*”

(No. 2)

“*It was a contradictory scenario that time. Why should I let other people take care of my child when he is ill? In other words, I felt guilty because I had to take care of other people’s children.*”

(No. 1)

### 3.3. Theme 3: Maternal Engagement

ICU nurses take on work and childcare responsibilities, even if it results in conflicting and difficult life experiences. ICU nurses with children must use coping strategies such as taking responsibility, becoming alert, seeking resources and support, and learning and practicing to achieve a family–work balance. These coping strategies, which are components of maternal engagement, are described below.

#### 3.3.1. Becoming Alert

Due to a lack of experience in caring for young children, nurse practitioners need to maintain vigilance, which also creates psychological pressure. Participant No. 8, who used to easily fall asleep, stated that the pressure of caring for the child at night made her alert when she would be sleepy. After work, she felt even more tired because of the parenting role.

“*If my child is nearby, he tends to wake up even by a slight noise, and then, I also wake up. Exactly! My husband also wondered why I became so alert. I used to fall asleep easily…*”

(No. 8)

Furthermore, a participant discussed the parenting pressures experienced by her:

“*…You are afraid that your child may get hurt. I feel that a child at such a young age is very active. As a result, you must always pay attention to your child.*”

(No. 3)

#### 3.3.2. Taking Responsibility

Although it is impossible to take care of a child the whole day because of work, the participants believed that the role of a mother should be embodied in the practical care work. Therefore, during time outside work, the participants tried to provide care and companionship in person to their children.

“*For example, yesterday was a holiday, so I let my son sit on my lap the whole day. I gently shook him and enjoyed interacting with him. I particularly enjoyed breastfeeding my child.*”

(No. 6)

After becoming mothers, the participants started to understand the importance of “responsibility” and motherhood. Even if other family members assisted in sharing the care work, they still believed that the parents should be the main educators, rather than relying on others, as described by the participant below:

“*Parents are the primary role models of children. Although grandparents can assist in childcare, they are not responsible for educating and raising children. Parenting and education actually fall on parents.*”

(No. 1)

Participants found that motherhood is embodied in the daily pragmatic parenting and care for children, and they gradually understood the responsibilities of motherhood and found that motherhood issues were constantly on their minds.

#### 3.3.3. Seeking Resources and Support

For ICU nurses who need to work swing shifts, it is necessary to seek support systems to assist in childcare, including support from family members, ICU colleagues and supervisors, and the use of peripheral resources.

“*If we experience any situations, as long as we tell the head nurse in advance, she will try her best to change shifts for us.*”

(No. 1)

“*understands that I have to work shifts and cannot take care of my child every day. Besides, my husband is also willing to take parental leave. He is willing to take care of the child and share the burden of parenting.*”

(No. 3)

“*My child had a fever due to adenovirus infection. At that time, I asked for a favor from the schoolteacher to take care of my child. I offered to pay a higher daily rate to help take care of my child for one day because I really needed to go to work that day. The agreed and helped me take care of my child for one day.*”

(No. 1)

Expressing milk at the workplace is a right of breastfeeding women. However, as the ICU requires teamwork, obtaining the assistance of colleagues from the unit can help breastfeeding women safely collect breastmilk during work. A participant mentioned that she felt sorry when her breastmilk collection increased the workload of her colleagues.

“*When I work evening and night shifts, there are more colleagues. They will pay attention to the timing for me to collect breastmilk, and then, urge me to do so. They are so sweet.*”

(No. 6)

#### 3.3.4. Keep Learning and Practicing

When faced with childcare-related issues, mothers need to spend time exploring and learning from their experiences. Even though the participants were nurse practitioners, they needed preparation and knowledge of motherhood.

“*When my son was infected with enterovirus, he had a fever and sore throat. He was unwilling to eat anything, so I fed him ice cream…Many people laughed at me: ‘You are a pediatric ICU nurse practitioner, how could you feed a very young infant ice cream?’ But my son could not eat anything, I had no choice. Eating ice cream was better than receiving IV(intravenous) infusion!*”

(No. 1)

Adjusting their original lifestyle and psychological self-transformation, such as by appropriate time allocation and staying calm when the baby is crying, helped nurse practitioners adapt to childcare life. The process of being a mother involves continuous learning and practicing for effective parenting.

“*My son is now 10 months old, and I think that I have gradually adapted myself to his lifestyle after month 9, such as knowing his physiological lifestyle, when he will wake up or have a poo…Therefore, I feel that I am getting used to his lifestyle now.*”

(No. 9)

Overall, the participants found that being alert, taking responsibility, seeking resources and support, and continuously learning and practicing helped them face the challenges of motherhood and establish family–work balance.

## 4. Discussion

The study results indicated that ICU nurses faced a dilemma due to their work and motherhood roles, resulting in a conflict between their family and work lives. They employ coping strategies, such as accepting responsibility, becoming alert, seeking resources and support, and learning and practicing, all of which are components of maternal engagement that aid in addressing the role of the mother. Although nursing is a predominantly female-dominated profession, women with this professional background should not be expected to navigate motherhood with ease. Contrarily, as a group, nurses are easily overlooked in the process of motherhood because of their nursing background and work.

Moreover, the study results highlighted the importance of paying attention to working women’s motherhood experiences. To facilitate discussion, we reviewed the implementation of the nursing-friendly workplace policy in Taiwan from 2011 to 2021 and compared it with the results of this study; additionally, we proposed improvement suggestions from two perspectives—policy and culture.

If both genders can share and balance each other’s family and work responsibilities, it will ease the pressure on mothers. Affected by various factors, such as social expectations and shaping parental role, novice mothers are usually direct participants in childcare, while novice fathers are indirect participants, which suggests that the burden of actual childcare is more on women than on men during the parenting process [4]. Two participants in this study stated that their spouses took leave without pay or played the role of a full-time caregiver at home to assist in childcare. This study found that, regardless of the extent of their partner’s parental role, a husband or partner is a great source of support for women adjusting to their family and work roles. Therefore, not only women but their partners also need to fulfill parental roles. Thus, encouraging fathers to engage in the role of fatherhood will help reduce the childcare pressure on working women.

Prenatal education and parent–child education courses should provide information to fathers in order to facilitate their understanding of the parenting process and their role as a father. In this way, gender consensus and mutual assistance in the parenting process can be achieved [18]. As nurses have different levels of expertise, they differ in their ability to provide childcare. Nurses who play the role of a caregiver at work can be easily stereotyped as having superior ability to perform their parenting role. Nursing personnel, who are obstetric caregivers, should avoid preconceived ideas and assess their neonatal care abilities to ensure that timely and appropriate assistance can be provided.

The participants in this study indicated that, although they could be expressing breastmilk at work, due to the fluctuating conditions of patients in the ICU, while expressing breastmilk, they worried about adversely affecting teamwork and burdening their colleagues, which created difficulty in implementing their work and motherhood roles. This result is similar to that of a previous study that investigated the breastfeeding experiences of nurses working swing shifts [19]. A review of past studies regarding the ranking of employee workplace health promotion issues found that, for both male and female employees, a breastfeeding-friendly environment was ranked last among workplace-friendly measures [20]. Compared with the results of this study, in the past 10 years, a breastfeeding-friendly environment has not been valued by all employees in Taiwan. Moreover, this study found that, for working women, there is still a lack of support for continuous breastfeeding, which is worthy of reflection and improvement. A supportive workplace atmosphere can help women adapt to motherhood and reduce their stress, and lower maternal pressure can improve mothers’ breastfeeding effectiveness [21].

Support from supervisors and colleagues, sufficient manpower, and attention to nursing personnel’s right to breastfeed are required to create a breastfeeding-friendly workplace environment [22]. Furthermore, the implementation of friendly workplace policies will rely on the support and creation of unit managers. Managers have a responsibility to create a friendly workplace environment for the employees [23]. The management in caring leadership is developed from caring and care, and the value reflecting human dignity is derived from the leader’s responsibility for humanization and cultural attitudes, as well as respect for the uniqueness of nurses. Caring leadership enables nurses to provide care with dignity, and only through consensus among leaders and subordinates can important issues be highlighted [24]. Nursing managers can take practical actions to support the motherhood identity of nurse practitioners in their unit. For example, when a nurse returns to the unit after childbirth, a nursing manager should consider the disease conditions of the patients assigned to her and coordinate with other colleagues to provide the required assistance. Furthermore, nursing managers should use caring leadership to create a friendly workplace atmosphere, which may indirectly reduce the possible incivility behavior between colleagues and enable nursing colleagues to support each other, reducing the conflict between nurses’ work and motherhood roles [25].

This study has several strengths and limitations. Viewing the mothering experience from a micro perspective can counter the insufficiency of cross-cultural research from a macro perspective. Therefore, rather than examining the mothering experience from the perspective of national policy and social culture, this study uses a qualitative research method to deeply understand the experience of mothering from the micro perspective of working women. The study participants belonged to a medical center in central Taiwan, however, the nursing manpower utilization and swing-shift system in other healthcare institutions and medical centers may be different. Therefore, the study results might not be inferred to the ICU nursing workplace conditions of other hospitals. Moreover, the study participants were ICU nurses whose workplace conditions are different from those of nurses in general wards. Thus, future studies should also investigate ward nurses’ opinions regarding a friendly workplace environment to better understand hospital nurses’ needs in this regard.

## 5. Conclusions

The study findings revealed that when ICU nurses resumed work after becoming mothers, breastmilk collection was the most challenging period for them, while balancing their work and motherhood roles. Furthermore, the adjustment required to strike a family–work balance continues to pressurize ICU nurses developing a motherhood identity. Through caring leadership, nursing managers can assist nurse practitioners developing a motherhood identity to better adjust to their work and family lives.

In the era of comprehensive and changing information, policy reforms and organizational restructuring continue to rely on teamwork. Nursing managers should create friendly nursing workplace environment and pay attention to the work–life balance of frontline nurses so that they can achieve better job satisfaction. In the future, healthcare organizations should develop plans and policies that focus on nurses’ working life quality and provide information regarding humanistic practices and policies to nursing management. Furthermore, nursing managers can gradually implement these policies, starting by creating a friendly workplace environment.

## Figures and Tables

**Table 1:** Participants’ demographic characteristics (*n* = 10).

No.	Age	Unit	Clinical Ladder	Educational Level	Number of Children	Age of Children	Provider of Childcare
1	37 years	NICU ^1^	N3	Master	1	18 months	Childcare center
2	31 years	ICU ^2^	N2	University	1	30 months	Parents
3	35 years	CCU ^3^	N2	University	1	15 months	Mother-in-law, Husband
4	35 years	ICU	N2	University	2	7 years, 3 years	Parents
5	28 years	ICU	N1	University	1	22 months	Mother-in-law
6	28 years	RICU ^4^	N1	University	1	4 months	Mother-in-law
7	34 years	TNCU ^5^	N3	University	2	3 years, 10 months	Foreign helper
8	34 years	SICU ^6^	N2	University	1	6 months	Husband
9	37 years	CCU	N3	University	1	10 months	Husband
10	33 years	ICU	N2	University	1	19 months	Nanny

Note: ^1^ NICU = Neonatal intensive care unit; ^2^ ICU = Intensive care unit; ^3^ CCU = Coronary care unit; ^4^ RICU = Respiratory intensive care unit; ^5^ TNCU = Trauma neuroscience care unit; ^6^ SICU = Surgical intensive care unit.

## Data Availability

The data presented in this study are available upon request from the corresponding author.
